# A systems biology approach to elucidating epigenetic regulation of cardiac hypertrophy

**DOI:** 10.1186/1471-2105-14-S17-A12

**Published:** 2013-10-22

**Authors:** Gipsy Majumdar, Rajendra Raghow

**Affiliations:** 1 Department of Rheumatology, University of Tennessee Health Science Center, Memphis, TN 38104, USA; 2 Department of Pharmacology, Veteran’s Affairs Medical Center, Memphis TN 38104, USA

## Background

Reversible acetylation of histone and non-histone proteins is involved in cardiac hypertrophy that may be attenuated by histone deacetylase inhibitors (HDACIs). Since the mechanistic underpinnings of how pan-HDACIs ameliorate pathological cardiac hypertrophy remain elusive, we investigated genome-wide consequences of HDACIs, trichostatin A (TSA) and m-carboxycinnamic acid bis-hydroxamide (CBHA), in Balb/c mice eliciting cardiac hypertrophy in response to interleukin-18 (IL-18).

## Materials and methods

To determine the contribution of the myocytes, uncontaminated by other cell types that populate the heart, we also undertook global gene expression analyses in H9c2 cardiac myocytes treated with either CBHA or TSA. Both HDACIs, regardless of the presence of IL-18, induced hyper-acetylation of cardiac chromatin that was accompanied with significantly changed gene expression profiles.

## Results

Thus, 184 and 147 cardiac genes were differentially expressed (DEGs) in in mice treated with IL-18+/-TSA and IL-18+/-CBHA, respectively; numerous DEGs were oppositely regulated by IL-18 and HDACIs. Exposure of cardiac myocytes to TSA for 6h and 24h led to differential expression of 468 and 231 genes, respectively. H9c2 cells treated with CBHA for 6h and 24h elicited 768 and 999 DEGs, respectively. We carried out *in silico* analyses of genes that were differentially regulated by IL-18, CBHA or TSA using Ingenuity Pathway Analysis (IPA), Kyoto Encyclopedia of Genes and Genomes (KEGG) and Core_TF programs. These data revealed that, in the heart, IL-18 treatment induced TNF-α and IFNγ specific gene networks that were connected with a repertoire of signaling kinases (PTEN-PI3K-AKT and MAPK) and transcription factors (Myc, p53, NFkB and HNF4A) known to control immunity and inflammation, cardiac metabolism and energetics, and cell proliferation and apoptosis. We present evidence to indicate that both HDACIs apparently counteracted the IL-18-induced signaling pathways and gene networks to allay the mechanisms of pathological cardiac hypertrophy. Specifically, we found that the expression of phosphatase and tensin homolog (PTEN) was induced by TSA and CBHA both of which suppressed IL-18-induced PI3K-AKT-MAPK signaling underlying pathological cardiac hypertrophy. Similarly, both CBHA and TSA also profoundly influenced the gene expression profiles in H9c2 cardiac myocytes treated in vitro.

## Conclusions

Initially (6h post-treatment), both HDACIs impinged on numerous genes associated with adipokine signaling, intracellular metabolism and energetics, and cell cycle and continued exposure for 24h to CBHA or TSA led to the emergence of a number of apoptosis- and inflammation-specific gene networks. We interpret these observations to mean that the anti-inflammatory and anti-proliferative actions of HDACIs are quite similar in the intact heart and are largely myocyte-intrinsic.

